# Acceptance and Commitment Therapy is feasible for people with acquired brain injury: A process evaluation of the BrainACT treatment

**DOI:** 10.1177/02692155231218813

**Published:** 2023-12-15

**Authors:** Johanne CC Rauwenhoff, Yvonne Bol, Frenk Peeters, Caroline M van Heugten

**Affiliations:** 1 School for Mental Health and Neuroscience, Faculty of Health, Medicine and Life Sciences, 5211 Maastricht University, Maastricht, the Netherlands; 2 School for Mental Health and Neuroscience, Department of Neuropsychology and Psychopharmacology, Limburg Brain Injury Centre, Maastricht, the Netherlands; 3 Department of Psychology, 8018 Norwegian University of Science and Technology, Trondheim, Norway; 4 Department of Clinical and Medical Psychology, Zuyderland Medical Centre, Sittard-Geleen/Heerlen, the Netherlands; 5 Department of Clinical Psychological Science, Faculty of Psychology and Neuroscience, 8018 Maastricht University, Maastricht, the Netherlands; 6 Department of Neuropsychology and Psychopharmacology, Faculty of Psychology and Neuroscience, 8018 Maastricht University, Maastricht, the Netherlands

**Keywords:** Acquired brain injury, Anxiety, Depression, Acceptance and commitment therapy, Process evaluation

## Abstract

**Objective:**

To evaluate the feasibility of Acceptance and Commitment Therapy for people with acquired brain injury.

**Design:**

A process evaluation of the BrainACT treatment was conducted alongside a randomised controlled trial.

**Setting:**

Psychology departments of hospitals and rehabilitation centres.

**Subjects:**

Tweny-seven participants with acquired brain injury and 11 therapists.

**Intervention:**

BrainACT is an Acceptance and Commitment Therapy adapted for the needs and possible cognitive deficits of people with acquired brain injury, provided in eight one-hour face-to-face or video-conference sessions.

**Measurements:**

The attendance and compliance rates, engagement, satisfaction, and perceived barriers and facilitators for delivery in clinical practice were investigated using semi-structured interviews with participants and therapists and therapy logs.

**Results:**

212 of the 216 sessions in total were attended and 534 of the 715 protocol elements across participants and sessions were delivered. Participants were motivated and engaged. Participants and therapists were satisfied with the intervention and participants reported to have implemented skills in their daily routines acquired during therapy. Key strengths are the structure provided with the bus of life metaphor, the experiential nature of the intervention, and the materials and homework. Participants and therapists often preferred face-to-face sessions, however, when needed video-conferencing is a good alternative.

**Conclusion:**

BrainACT is a feasible intervention for people with anxiety and depressive symptoms following acquired brain injury. However, when the content of the intervention is too extensive, we recommend adding two extra sessions.

## Introduction

In this study, we present the outcomes of the process evaluation of the BrainACT intervention. The process evaluation was conducted alongside a trial,^1^ among participants allocated to the Acceptance and Commitment Therapy arm of this trial. The BrainACT study is a randomised controlled trial in which the effectiveness of an adapted Acceptance and Commitment Therapy for anxiety and depressive symptoms following acquired brain injury is investigated. Next to investigating the effectiveness of an intervention, it is important to monitor the feasibility, implementation and delivery of the intervention in a systematic way, as this will enrich the interpretation of the results.^2^ In addition, next to quantitative data on effectiveness, process evaluations, through qualitative data, can provide narratives on implementation processes, their nuances and the identification of complex processes, which are not identified with questionnaires.^3^

Previous studies have shown the potential effectiveness of Acceptance and Commitment Therapy for people with brain injury-related mood symptoms.^4–6^ Acceptance and Commitment Therapy is a third-wave behavioural therapy, which focuses on the improvement of psychological flexibility. This entails living in alignment with personal values while accepting internal processes and being in contact with the present moment.^7^ We developed the BrainACT intervention, which is a treatment programme adapted for the needs and possible cognitive deficits of people with brain injury and investigated its effectiveness in a series of four single cases.^8^ Despite promising results in terms of effectiveness, no process evaluation has been conducted on the feasibility of Acceptance and Commitment Therapy for people with acquired brain injury.

The current process evaluation was performed before the analysis of the effectiveness to avoid bias in the interpretation of results.^2^ The feasibility of the BrainACT intervention, provided face-to-face as well as through video-conferencing, was evaluated by: (1) the attendance (number of sessions attended by participants) and compliance rates (elements of the protocol delivered by therapists); (2) engagement with the protocol; (3) satisfaction of participants and therapists; and (4) barriers and facilitators for delivery in clinical practice as experienced by both participants and therapists.

## Method

Recruitment of participants for the BrainACT study took place between April 2019 and January 2022 at medical psychology and rehabilitation departments throughout the Netherlands. All participants who were randomised into the Acceptance and Commitment Therapy arm of the BrainACT trial were eligible to participate in this process evaluation. Ethics approval for the BrainACT study has been given by the medical research ethics committee of Maastricht University Medical Centre and Maastricht University (committee reference number NL65349.068.18). All participants gave informed consent. The study protocol is performed in accordance with the relevant guidelines and published elsewhere.^1^ The study is registered in the Dutch Trial Register (now Clinical Trial Registry Platform), NL691, NTR 7111. Registered on 26/03/2018. https://trialsearch.who.int/Trial2.aspx?TrialID = NTR7111

The BrainACT intervention is described in detail elsewhere.^9^ An overview of the intervention is given in Supplementary Table 1.

Patients who received the BrainACT treatment and therapists who delivered the treatment in the trial were invited to participate in this process evaluation. After each treatment session, the therapists filled in the therapy logs. Here, they could indicate the elements (different metaphors or exercises) of the protocol that were covered during the session and possible deviations from the protocol. Furthermore, they registered how long the session lasted and if the participant completed the homework assignments. This was rated on a scale from 1 (did no homework) to 5 (did all homework assignments). Lastly, therapists rated their own Acceptance and Commitment mode on a scale from 1 to 10, ranging from not being in the mode to being in a good mode. Examples of being in a good Acceptance and Commitment mode include the use of self-disclosure, being mindful, and naming their own fusion during a session.

The engagement with the protocol, satisfaction of participants and therapists, and perceived barriers and facilitators for delivery in clinical practice were investigated using semi-structured interviews. Interviews were performed through telephone calls by research assistants who were not involved in the development of the protocol or effectiveness trial of the intervention. The interviews were audio-recorded and transcribed verbatim. Participants were asked if they benefited from the treatment, if the treatment helped them to cope better with (negative) thoughts and emotions, what they benefited the most from and the least (what could be improved), if the treatment changed anything in their lives, and if they would recommend the treatment to other people with brain injury.

Therapists were asked how they experienced working with the BrainACT protocol, if the treatment went as expected, how the motivation of the participants was, if they thought the participant benefited from the treatment, and if the adjustments for people with brain injury were sufficient. Furthermore, their opinion was asked on the materials (workbook and manual), the exercises and metaphors, planning of the sessions, and homework.

Both participants and therapists were asked how satisfied they were with the treatment and to rate the treatment on a scale from 1 to 10 (1 being bad and 10 being very good). Lastly, if participants or therapists received or provided the treatment through video-conferencing they were asked about their experience and the benefits and disadvantages of treatment by means of video-conferencing. Table 1 describes the measurement instruments used in this process evaluation.

**Table 1.** Outcome measures and measurement instruments of the process evaluation.

	Therapy log	Interview
**Attendance rate**	Therapists indicated when the session took place and how long they lasted.	–
**Compliance rate**	Therapists checked which elements of the protocol were covered (from which percentages were calculated) and indicated if they added components to the intervention	–
**Engagement with the protocol**	Therapists indicated whether participants completed their homework on a scale from 1 to 5.	Therapists were asked if participants were motivated and participants were asked about the use of Acceptance and Commitment Therapy skills after finishing the intervention.
**Satisfaction of participants and therapists**	–	Participants and therapists were asked to score the intervention from 1 to 10 and elaborate on the grade.
**Perceived barriers and facilitators**	–	Therapists and participants named barriers and facilitators.
**Video-conferencing**	–	Therapists and participants were asked about their experiences delivering and receiving the treatment through video-conferencing.

The quantitative data were analysed with descriptive statistics using SPSS, version 25. The compliance rate was calculated using the therapy logs, by describing the number of elements and calculating percentages based on how many treatment elements the therapists covered. Qualitative data from the semi-structured interviews with the participants and therapists were classified into categories by author JR, based on the contents of the answers. Quotes from the interviews are included to illustrate these categories.

## Results

In total, 27 out of 36 participants allocated to the BrainACT treatment, participated in the semi-structured interview. Information on the participants can be found in Table 2. The average age of the 11 therapists was 42.5 (SD 9.0) and nine were female. On average, they had 15.8 (SD 8.3) years of experience in working with people with brain injury and 5.6 (SD 2.7) years with Acceptance and Commitment Therapy. Therapists worked at seven of the 11 participating sites in the BrainACT trial.

**Table 2.** Demographic characteristics of the participants.

Demographic variables	Mean (SD) or *n* (%)
Sex, *n* women (%)	12 (44.4)
Age, mean (SD)	52.3 (13.0)
Marital status, *n* (%)	
Married	12 (44.4)
Unmarried	7 (25.9)
Living together with partner	7 (25.9)
Divorced	1 (3.7)
Education, *n* (%)	
No education	1 (3.7)
Low (primary education and lower vocational education)	5 (18.5)
Medium (general secondary education and secondary vocational education)	9 (33.3)
High (pre-university education, higher professional education and university education)	12 (44.4)
Employment, *n* (%)	
Incapacitated for work	11 (40.7)
Employed	10 (37.0)
Retired	3 (11.1)
Unemployed	2 (7.4)
Other	1 (3.7)
Brain injury-related variables Mean (SD) or *n* (%)	
Time (months) since injury, mean (SD)	18.9 (24.0)
Type of injury, *n* (%)	
Ischemic stroke	15 (55.6)
Hemorrhagic stroke	7 (25.9)
Traumatic brain injury	5 (18.2)
Stroke location, *n* (%)	
Left hemisphere	8 (36.4)
Right hemisphere	9 (40.9)
Subarachnoid space	2 (9.1)
Cerebellum	1 (4.5)
Multiple locations	1 (4.5)
Unknown	1 (4.5)
Severity traumatic brain injury	
Glasgow Coma Scale score, mean (SD)	10.7 (5.9)

Four out of the 216 sessions were not attended (212 or 98.2% attended). In total, over all sessions and participants, 534 of the 715 elements of the protocol were delivered (75% compliance, ranging from 47% to 100% per participant). In 29 of the 216 sessions (13%), therapists added one or more exercises or metaphors not included in the protocol. The sessions in which therapists most often added elements were the sessions of defusion and the self. The metaphor which was most added was the ‘thinking machine’ which is a defusion metaphor to illustrate that our mind produces a constant stream of thoughts. The average time spent on a BrainACT session was 58.1 min (SD = 7.3).

Therapists could choose the order of sessions per participant. The most used sequence of sessions (in 18 out 27 therapy trajectories) was: values, committed action and mindfulness, effect of control, acceptance, defusion, self-as-context, defusion and mindfulness, and psychological flexibility. The second most used sequence (in 3 out of 27 therapy trajectories) was: effect of control, acceptance, values, committed action and mindfulness, defusion, defusion and mindfulness, self-as-context, and psychological flexibility. Other sequences were only used once or twice.

The average Acceptance and Commitment mode of the therapists was 7.3 (SD = 1.0) as scored on a scale from 1 to 10. Table 3 shows the compliance rate, session length, and therapy mode per session based on the therapy logs. Of the 27 participants, ten received the intervention (partly) through video-conferencing.

**Table 3.** Elements of the BrainACT protocol delivered and average minutes per session based on the therapy logs.

Session	Compliance rate: protocol elements delivered (%)	Mean session length; min (SD)	Mean therapy mode therapist; scale 1–10 (SD)
Values	89/108 (82%)	63.3 (14.9)	7.3 (1.2)
Committed action and mindfulness	63/81 (78%)	57.0 (15.1)	7.4 (1.0)
Effect of control	64/78 (82%)	57.2 (7.9)	7.4 (1.6)
Acceptance	65/81 (80%)	56.9 (8.5)	6.8 (1.6)
Defusion	85/135 (63%)	57.0 (7.0)	7.3 (1.5)
The self-as-context	24/26 (93%)	57.7 (8.7)	7.4 (1.4)
Defusion and mindfulness	55/81 (68%)	54.2 (6.9)	7.0 (1.4)
Psychological flexibility	89/125 (71%)	60.6 (12.7)	7.7 (1.1)

Five therapists mentioned that the participants they treated were very motivated, three indicated that the motivation was good, and two therapists indicated that the motivation of the participant they treated varied per session.

On a numeric scale from 1 (made no homework) to 5 (made all homework assignments), the average score for making homework was 4 (SD = 0.8).

### Acceptance

Out of the 27 participants, 26 answered that the therapy helped them to cope better with (negative) emotions. However, seven of these participants placed a side note that it was difficult to sustain the acquired skills when the therapy was over or that they did not manage to always cope better with emotions. Acceptance was mentioned as an important part of the outcome of the intervention by 22 out of 27 participants. One of the participants said:

‘*It has helped me to give everything a place in my head. I can cope better with the brain injury and I am no longer depressed by its effects. I experience more space in my mind*.’

Furthermore, participants stated they have accepted that certain things are not possible anymore and therefore feel less frustrated when certain tasks do not go as planned. Finally, participants mentioned that therapy helped them to accept emotions related to the brain injury, such as sadness, loss, frustration or anxiety about a stroke recurrence. As an example:

‘*I would say that it did help me to turn around or accept certain thoughts. I've always had a big fear that I would have another brain infarct, which would end badly. It has happened three times now and I was lucky every time. The fear is that it will not end well the next time. I have accepted that more … The fear was certainly present, but it has reduced because I have accepted it more. It is not completely gone, but if you keep putting it away, it comes back twice as hard*.’

### Defusion

Out of the 27 participants, 24 answered that the therapy helped them to cope better with (negative) thoughts. Several patients said that negative thoughts are still present but have less impact. Furthermore, it was mentioned that the idea that ‘thoughts are just thoughts and might not necessarily be true’ helped them a lot. For instance:

‘*Certainly, the treatment increased the awareness of the negative emotions and thoughts, which are still present. Even if the end-result is not satisfactory, you learn to accept it better. By thinking more about what you are actually worried about, you can better focus on important things. I try to keep this line of thinking in my mind*.’

### Values

Ten out of 27 participants mentioned values as an important part of the intervention. They said that it was good to think about and act on the things that are important in life. Some people mentioned that they changed hobbies or see their family more. Furthermore, patients stated that their values help them make decisions when their energy level does not allow them to do everything they want. One of the participants said:

‘*I think the part of determining the values and acting on the values helped me the most. Now I'm very conscious about what I'm going to do over the weekend: when do I have time, what do I want to do, who am I going to do something with: then I look at what is important. This also came back with the measuring moments, how have you acted on your value lately? That did make me aware again that I had to pay more attention to my husband, for example. That insight helped*.’

### Mindfulness

Nine out of 27 participants mentioned that they still use mindfulness exercises or that they live more in the present moment, which was illustrated as follows:

‘*I live much more in the here and now. Before, I was very much preoccupied with the past and thinking about the future. I still have good days and bad days, but now I can enjoy the good days more*.’

### Self

Two participants indicated changes regarding the self or self-image. It was mentioned that it is reassuring to not have to fulfil their expectations of themselves. Furthermore, one person mentioned a stronger connection to the observing self:

‘*Yes, ACT changed something very big for me. I used to say that I am not the same person anymore, but because of ACT, I know that I am still the same person and I have not changed. Only my behaviour is different because of my disability, but I am still the same person. That is the biggest and most important change for me*.’

### Satisfaction

The participants rated the intervention with an average of 7.8 (SD = 0.6). Most participants (25 out of 27) were satisfied with the treatment. The two participants that were not satisfied found the therapy too difficult or stated that the therapy did not solve their problems.

All participants indicated that they benefited from the treatment, in one way or another. Furthermore, all participants said they would recommend the treatment to other people with a brain injury and one participant already recommended the therapy to a friend who suffered from an ischemic stroke. Several participants (6 out of 27) also mentioned that the therapy would not only be helpful for people with a brain injury but that everybody could benefit from the therapy.

The average score the therapists gave the intervention was 8 (SD = 0.6). Nine therapists indicated that they thought the participants they treated benefited from the treatment. One therapist said:

‘*I think it is a really nice protocol for people with brain injuries and I really hope that it will become a specific module for this target group*.’

Two therapists thought it was difficult to assess if the participant benefited from the treatment.

All 11 therapists indicated that the BrainACT intervention was sufficiently adapted for people with brain injury.

## Barriers and facilitators, which patients named throughout the interviews were categorised

### Facilitators

Participants (8 out of 27) stated they benefited from the structured nature of the intervention. Additionally, 7 out of 27 participants mentioned that the workbook and homework were clear. Several patients said that it was helpful that all the sessions had a clear theme, that the coherence between the sessions was clear, and that in the end, everything came together. Furthermore, several patients mentioned the bus of life metaphor helped to provide a clear structure. The bus of life metaphor is explained in Box 1.

‘*The part of the bus helped me the most. I don't use it anymore, because I have been able to give it a place. I can't say anything that hasn't helped, it's the whole concept that helps, which of course started with the bus trip and all the parts which are connected to that. They all made their own contribution to giving it [the consequences of the brain injury] a place*.’

‘*The bus [metaphor] made an impression on me. It kept coming back, therefor I could remember it and because of that it made sense to me*.’

Several participants (12 out of 27) reported different experiential exercises or metaphors that made an impact on them and were seen as insightful. Furthermore, it was said that the metaphors helped to understand the processes and helped to remember them, especially the ‘bus of life’ metaphor and the metaphor ‘thug-of-war with a monster’ (explained in Box 1) were named multiple times in this regard.

‘*Sometimes I found the exercises really confronting, like the monster, but that was also a real eye-opener*.’

Box 1. The bus of life metaphor is used to explain the different core components of Acceptance and Commitment Therapy.

In the session on values, we reflected on where you want to go (values) and what you need to do to make the bus move in that direction (actions/next stop). After brain injury, the bus and the condition of the road might have changed. For example, it moves slower or there are more bumps in the road (you are more easily distracted or tired). But the direction your bus is going (what you value and care about) remains the same!

As you begin driving in that valuable direction, you pass several stops. At those stops, passengers get on. The passengers represent your thoughts, feelings and bodily sensations. And as you are driving, these passengers begin to interfere with the ride. ‘Go left here, go right here, stop, go back!’ Some will become very annoying, trying to get your attention and influence the journey.

During the session effect of control, we considered what happens when you try to get rid of your passengers. If you try your best, you can work them off your bus for a while, giving you some peace of mind. You will find, however, that it quickly gets around that there is a bus driver trying to get rid of annoying passengers and soon your bus is full of passengers eager to fight with you.

In the session on acceptance, we learned how to deal with these annoying passengers. Instead of spending all the time trying to work them off your bus, or obeying them so they don't get even more annoying, you learn to give space to these passengers without having to do anything with them. The passengers get to just sit on the bus while you steer your bus in the direction that is valuable to you.

In the session on defusion, you learned how to distance yourself from all the thoughts you have. Your passengers are yelling all sorts of things at you (your thoughts). As a bus driver, you don't want to be swept away by this stream of commands. Nor do you want to simply obey them. You want to learn how to register them from a distance. ‘I heard you passengers, thanks for the feedback!’ You then choose to keep moving in the direction that is important to you.

In the session on the self, we learned how to see that we are not our thoughts and feelings, besides we just are. The bus is not only made up of all the passengers (which sometimes seems that way when it is so packed), you as the bus driver are also still there! You will always be the driver of your own bus and the passengers on your trip will be getting on and off. Imagine you are sitting in a bus shelter and your bus passes by. What do you see? We also dwelled on your self-image and what you expect of yourself. It could be that you are stuck in the idea that you have to be the best bus driver and you try your very best to live up to this expectation. Before you know it you are driving in a non-valuable direction.

While you are driving, it is important to be consciously aware of everything that you experience at that moment, both in your inner- and outer world. As a bus driver, you do not want to mindlessly drive around. You want to pay attention to what you are doing and give space to what you are experiencing at that moment; a nice song on the radio, the sunshine etc. During the session here & now, we practised this skill. We also did an exercise almost every session to bring our attention to the here & now.

We have encountered the bus many times during the treatment. It, therefore, indicates our journey through this treatment. You can clearly see how the different ACT skills combine to allow you to fill in your life based on the things you care about, along with the problems you are experiencing. The acceptance piece of ACT helps you to not get caught up in a fight with your own unpleasant experiences. This gives you more space for the things that are really important to you, the commitment piece. 
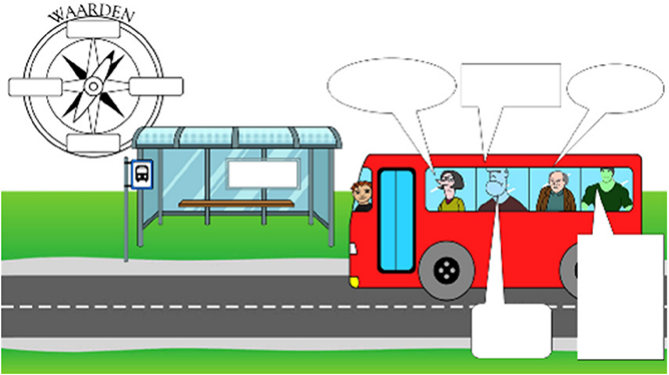

Box 2. Tug-of-war with a monster

The situation you are in now is a bit like the following:

Imagine you are playing tug-of-war with a monster. The monster represents all your misery: your brain injury, your pain, your grief, your physical symptoms. It is big, ugly and very strong. Between you and the monster, there is a big gaping hole to which you are slowly being pulled. If you lose the tug of war you will fall over the edge and not much will be left of you. So you pull and pull to avoid this because you don't want the pain and all your misery. But the monster is bigger and stronger than you. The harder you pull, the harder the monster pulls and the closer you get to the edge of the ravine.

It is difficult to see if you are fighting this hard to not fall into the hole, but you don't have to win the tug-of-war. What could you also do? Let go of the rope.

### Barriers

Four patients felt that the protocol left little room to talk about the things that troubled them at that time.

*‘A lot of things were planned and framed so there was sometimes less time to discuss things that I needed.’*

Additionally, not everyone liked the metaphors, one participant mentioned:

‘*I found the metaphors about my own feelings difficult because I don't express my feelings that easily. So I found it hard to be concrete about my feelings*.’

## Barriers and facilitators, which therapists named throughout the interviews were categorised

### Facilitators

Seven of the 11 therapists mentioned the bus of life metaphor as a helpful component as it is the common thread in the therapy. Several therapists reported that the use of a small number of metaphors and the repetition of the bus of life metaphors increased the retainment.

Furthermore, several therapists mentioned that they liked the experiential nature of the protocol. The exercises helped participants to experience the ACT processes.

‘*Well, I really enjoyed it. It was a very nice programme. I found it practical and simplified, and I also found it rather experiential. There are exercises that can give a lot to think about with very few words*.’

Eight therapists mentioned they liked the materials and how the working book for the participant was structured.

### Barriers

Seven out of the 11 therapists experienced time constraints. They were unable to cover all the aspects of the protocol during the therapy sessions or felt pressured to complete everything on time or they did not cover all the aspects of the protocol.

Several therapists suggested that the content of the intervention should stay the same, as there were no elements that could be eliminated, but to add more sessions. Furthermore, five therapists mentioned that it was a challenge for them to work with such a structured protocol, as they are used to a more flexible way of treating or used to their own exercises and metaphors. It was also mentioned that the working book on certain spots contained too much text and could be simplified. Facilitators and barriers are summarised in Table 4.

**Table 4.** Facilitators and barriers according to participants and therapists.

	Facilitators	Barriers
Participants	Structure and nature of the intervention were beneficial	Not enough room to talk about current worries
	Clear workbook and homework	
	Session themes were helpful and gave coherence	
	Bus of life metaphor is helpful for most people in understanding and remembering	Metaphors are not helpful to some people to express their feelings
	Experiential exercises and metaphors are insightful	
Therapists	Bus of life metaphor was helpful as a common thread	
	Small number of metaphors and the repetition were beneficial	
	Experiential exercises were appreciated	Some therapists like to use their own exercises
	Intervention materials and workbook for participants were appreciated	Content should remain the same but the number of sessions needs to be increased. Workbook could be simplified.
		Time constraints were experienced due to the many elements of the protocol
		The structured protocol can hinder a more flexible way of offering therapy

### Video-Conferencing

Ten out of the 27 participants received the treatment (partly) through video-conferencing during COVID-19 lockdowns. They mentioned both facilitators and barriers of these online sessions. Several participants mentioned that online therapy saved them the commute to the hospital and therefore saved them energy. However, looking at a screen for an hour was too tiring and they were more easily distracted. Furthermore, someone who received both online and face-to-face sessions said that the face-to-face sessions made more impact and therefore were easier to remember. The barrier mentioned the most was that it was more difficult to connect with the therapist.

Several participants also mentioned that it was helpful to meet the therapist before the start of the intervention or to do the first session face-to-face. Moreover, some patients preferred therapy sessions through video-conferencing, while others preferred face-to-face sessions.

Four therapists delivered the BrainACT protocol (partly) through video-conferencing. The therapists recognised the advantage for patients not having to travel to the clinic. Two therapists reported that the video sessions gave interesting leads for the therapy because the participant was in the context of their own living situation. Barriers that were named were increased distractibility (by for instance pets or housemates), several elements were more difficult to execute through video-conferencing (such as the pulling the rope with a monster), sessions being more tiring for both participants and therapist (often sessions were shortened), technical difficulties, participants being less open, and not being able to make eye contact.

## Discussion

This study showed that the therapists delivered the BrainACT intervention largely according to protocol and the participants attended most of the sessions. Participants seemed motivated and to have engaged well with the protocol during therapy and completed the homework assignments. Overall, both participants and therapists were satisfied with the intervention. Some participants explicitly appreciated the structured nature of the therapy, the experiential exercises and use of metaphors.

The attendance rate of the sessions was high, and most of the protocol elements (75%) were delivered as intended. This is lower than the 80%, which is often applied as a sufficient compliance rate.^2,10,11^ However, it is higher compared to a study investigating Acceptance and Commitment Therapy for cancer survivors (70%),^12^ but lower than a study examining this therapy for people with chronic lower back pain (80%).^13^ The somewhat low compliance rate is likely due to the fact that therapists thought that the content of the sessions was often too much and therefore they did not cover all the elements. This was especially the case concerning interventions delivered through video-conferencing. These sessions were often shorter since participants were more easily fatigued. Another explanation might be that therapists sometimes used metaphors or exercises they were more familiar with or found more appropriate for the participant. This was also observed by Clarke and colleagues.^12^ In a clinical setting, Acceptance and Commitment Therapy is often delivered in a flexible way. In the current protocol, several flexible elements were integrated into the treatment. These include optional metaphors and exercises and a flexible order of the sessions. The need for adaptable and modifiable treatments in clinical settings forms a challenge when implementing evidence-based treatment protocols in clinical practice.^14^

Following the intervention, most participants reported to continue using the therapy skills. The benefits of psychotherapy are extended when patients continue to use skills obtained during psychotherapy.^15^ Due to cognitive deficits, this might be more challenging for people with brain injury, therefore, booster sessions might help to rehearse and practice the skills for some individuals.^16^ The theme of acceptance was mentioned most often by participants and seems to be a key component and outcome of the intervention which aligns with the result of a study into experiences of stroke survivors following a didactic ACT intervention.^17^

Key strengths of the BrainACT intervention are the experiential nature of the intervention, the simplified materials of the workbook and homework, which were experienced as clear, and the structured nature of the protocol with help of the bus of life metaphor. It emphasises the importance of not using too many different metaphors at the same time and providing a clear structure within the therapy sessions and treatment as a whole. A clear barrier identified by the therapists was that the content of the sessions was sometimes experienced as too much. Therefore, therapists often skipped certain elements of the protocol. This seemed sufficient since none of the participants said that the content was too much and only one participant indicated that the intervention was too difficult.

Some strengths and weaknesses of this process evaluation should be mentioned. A strength of the study is that the results of the process evaluation were analysed before the results of the study into the clinical and cost-effectiveness of the intervention were known. Additionally, both participants and therapists were involved in the evaluation. The evaluation was performed independently of the intervention developers and researchers.

A weakness of the study was that part of the study was performed during the COVID-19 lockdowns, when the treatment was provided through video-conferencing which was not foreseen. Generally, participants seemed happy to receive help during this period but often would have preferred face-to-face sessions. Previous studies found that technology-based interventions for anxiety and depression following a stroke can be feasible.^18,19^ The barrier most often named by participants was that it was more difficult to connect with the therapist. Also, therapists had the feeling participants were less open compared to face-to-face sessions which is in line with previous studies that concluded that therapists found it difficult to communicate emotions and empathy and to connect with the patients online.^20^ Furthermore, video-conferencing was experienced as more burdensome and participants were more easily fatigued and distracted. This calls for some adaptations when providing the BrainACT intervention through video-conferencing.

Overall, both participants and therapists were satisfied with the intervention, giving it high grades. All participants would recommend the intervention to other people with brain injury and all therapists thought the intervention was sufficiently adapted for patients with brain injury.

This process evaluation has shown that the BrainACT intervention is feasible. Nevertheless, based on this process evaluation some recommendations for further improvement can be made. For participants for whom the content of the sessions is too extensive, we recommend adding extra sessions. Ten sessions would most likely be sufficient to complete the elements without time pressure. When it is possible to provide the intervention face-to-face this would be the preferred format, however, when needed video-conferencing is a good alternative. If doing so, it is recommended to deliver the first sessions face-to-face. The current study identified several key elements of the intervention, which should be part of the BrainACT protocol regardless of individual adaptations, such as the experiential exercises, the ‘bus of life’ metaphor, and the structured workbook.

In conclusion, BrainACT is a feasible individual intervention for people with brain injury experiencing depressive and anxiety symptoms.

**Clinical Messages**

- BrainACT, Acceptance and Commitment Therapy adapted for people with brain injury, is feasible for people with anxiety and depressive symptoms following acquired brain injury*.* Face-to-face delivery is preferred.
- Key strengths of BrainACT are the structure provided with the bus of life metaphor, experiential nature of the intervention and the simplified workbook and homework.

## Supplemental Material

sj-docx-1-cre-10.1177_02692155231218813 - Supplemental material for Acceptance and Commitment Therapy is feasible for people with acquired brain injury: A process evaluation of the BrainACT treatment Supplemental material, sj-docx-1-cre-10.1177_02692155231218813 for Acceptance and Commitment Therapy is feasible for people with acquired brain injury: A process evaluation of the BrainACT treatment by Johanne CC Rauwenhoff, Yvonne Bol, Frenk Peeters, and Caroline M van Heugten in Clinical Rehabilitation
