# Supplementary material for: Acceptance and Commitment Therapy is feasible for people with acquired brain injury: A process evaluation of the BrainACT treatment
Source: Clin Rehabil. 2023 Dec 15;38(4):530–42. doi: 10.1177/02692155231218813 (PMC10898201; doi:10.1177/02692155231218813)
Supplement: sj-docx-1-cre-10.1177_02692155231218813 - Supplemental material for Acceptance and Commitment Therapy is feasible for people with acquired brain injury: A process evaluation of the BrainACT treatment [file sj-docx-1-cre-10.1177_02692155231218813.docx]

| **Supplementary table 1**. Overview of the BrainACT intervention | | | |
| --- | --- | --- | --- |
| **Session title** | **Content** | **(Experiential) exercises and metaphors during the session** | **Homework exercises** |
| Values | Value exploration and defining core values. | - ‘bus of life’ metaphor  - Values sorting exercise  - Describing your gravestone or 80th birthday exercise  - Writing down core values | - Reading or listening to the summary of this session  - Explore which values deserver more or less attention  - Valuable pictures exercise |
| Action and Mindfulness | Committed action in the long and short term in relation to values. Education about mindfulness, and practising contact with the present moment. | - What's the next stop of your bus? (defining short term goals)  - Keep driving (defining long-term goals)  - Introduction mindfulness: raisin exercise | - Reading or listening to the summary of this session  - Perform a daily activity with full attention  - “valuable activity of the week”, performing one concrete action that fits within one of the patient's values  - Defining obstacles while making these homework exercises |
| Effect of control | Creative hopelessness; the undeniability of human suffering and the consequences in the long term of trying to control it. Defining strategies used to cope with negative thoughts and emotions. | - Mindfulness exercise: mindful breathing  - Identifying how the patient copes with unpleasant experiences.  - Tug-of war with a Monster or bouncing ball metaphor | - Reading or listening to the summary of this session  - Keeping track of unpleasant thoughts or feelings and how they coped with them  - Reread the metaphor of the monster or bouncing ball |
| Acceptance | Introducing acceptance as an alternative to control. | - Tug-of war with a monster, unwanted guest in your ‘bus of life’, or the finger trap metaphor  - Explanation on the difference between pain and suffering with the glass of water exercise  - Mindfulness exercise: making space and allowing what is there | - Reading or listening to the summary of this session  - Willingness exercise |
| Defusion (skill or technique to get some distance from one’s thought and feelings) | Changing the relationship with thoughts, naming the mind. | Mindfulness exercise: Attention for thoughts  - The passengers in your ‘bus of life’ (post-its)  - Naming the mind  - Singing difficult thoughts  - Mindfulness exercise: floating leaves on a river | - Reading or listening to the summary of this session.  - Watch the YouTube video of the ‘bus of life’  - Practice defusion exercises |
| The Self | Changing the relationship with thoughts about oneself and introducing the constant self. | - Negative and positive labels of oneself  - Explanation on the constant self  - Mindfulness exercise: mindful movement  - Suits (thoughts about oneself) that don’t fit metaphor | - Reading or listening to the summary of this session  - Take off a suit that doesn’t fit (anymore)  - Practice a mindfulness exercise |
| Defusion and Mindfulness | Repetition on defusion and mindfulness. | - Mindfulness exercise: the body scan  - Defusion exercises: physicalizing the thought  - Mindfulness exercise: mindful breathing | - Reading or listening to the summary of this session  - Practice a mindfulness exercise daily |
| Resilience | Review of the different core components, explanation on how these skills together lead to psychological flexibility, and preparation on relapse and setbacks. | - Car metaphor  - ‘bus of life’ metaphor and exercise  - Strategies on how to keep using acquired ACT skills in daily life  - Mindfulness exercise: mindful listening | - Reading or listening to the summary of this session  - Try to keep practising with the different skills  - Start integrating Acceptance and Commitment skills into your daily life  - Live your life as it is valuable to you! Stop fighting, start living! |
